# Experiential expertise in the co‐development of social and health‐care services: Self‐promotion and self‐dismissal as interactional strategies

**DOI:** 10.1111/1467-9566.13457

**Published:** 2022-03-30

**Authors:** Elina Weiste, Melisa Stevanovic, Lise‐Lotte Uusitalo

**Affiliations:** ^1^ 3860 Finnish Institute of Occupational Health Helsinki Finland; ^2^ 7840 Tampere University Tampere Finland

**Keywords:** client involvement, co‐development, decision‐making, experience knowledge, expertise, social and health care

## Abstract

Increasing client involvement in the development of social and health‐care services has resulted in clients being invited to present their experiential knowledge in service co‐development groups. Nevertheless, research has shown that their opportunities to really contribute to actual decision‐making are limited. This article investigates how client representatives initiate turns‐at‐talk in the decision‐making context and the way in which professionals respond to them. Using conversation analysis, we analyzed 15 h of recorded interactions in five co‐development workshops. Our data exhibited a systematic pattern that linked client representatives’ self‐promoting and self‐dismissive turns‐at‐talk to specific types of responses from professionals. When the client representatives highlighted the relevance of their experiential knowledge for making decisions, the professionals disregarded their contributions. However, if instead, the client representatives cast their experiential knowledge as irrelevant to the decision‐making activity at hand, the professionals subsequently appreciated this knowledge. Thus, paradoxically, in order to establish the relevance of their views, client representatives diminished their positions as experiential experts.

## INTRODUCTION

Recent decades have witnessed constant changes in the role of clients in social and health‐care services, which has transformed the perception and appreciation of professional and lay knowledge. Clients’ expertise, originating from their first‐hand experiences of illnesses and their treatment in health‐care and social service systems, has become essential in the planning, development and evaluation of these services (Crawford et al., 2002). Clients’ participation in their own care, as well as in the development of services, is deeply anchored in the idea that service users should participate in decision‐making processes (Thompson, 2007). Participation occurs in and through shared decision‐making (SDM) when professionals and clients interact in a dialogical, reciprocal relationship (Thompson, 2007, 1297). Shared decision‐making is thus seen as a collaborative process in which both the professional and the client are engaged in sharing information based on their professional and lay expertise (Charles et al., 1997). Clients are believed to have essential experiential knowledge of service processes and to best know their service needs (Charles et al., 1997).

The roles of experiential expertise on the one hand, and professional expertise on the other, have long been discussed in medical sociology. The shift to taking experiential knowledge into account took place around the 1980s, when Tuckett and colleagues’ (1985) suggested that if patients arrived at the consultation with their own conscious ideas about medical information and recommendations, the physicians’ ideas would remain separate from those of the clients, and the gap would persist throughout the consultation. This often unexplained mismatch between professional and lay ideas was seen to lead to failure in mutual communication (Tuckett et al., 1985). Habermas (1987, see also Prior, 2003) argued that expert culture had become essentially antidemocratic, and that movement towards increasing client involvement called for the democratization of knowledge. At a professional level, this led to the democratization of decision‐making procedures by encouraging client participation (Charles et al., 1997). Clients were considered to possess knowledge relevant to decision‐making, such as knowledge about their own bodies, illnesses and the ways in which various treatments affected their bodies and lives (e.g. Hibbert et al., 2002). This knowledge was considered valuable when trying to make services more responsive to clients’ needs (Crawford et al., 2002). In this respect, clients came to be considered experts by virtue of their experience (Prior, 2003) and were expected to possess a significantly different type of knowledge of their illnesses to that of professionals (Jones & Pietilä, 2020).

Although social and health‐care service clients are thus assigned an increasingly active role as experts with the right to participate in service development alongside professionals (Thompson, 2007), studies have indicated that their ability to contribute to actual decision‐making is surprisingly limited (e.g. Snyder, 2014). For instance, when clients in co‐development workshops analyzed by Weiste et al. (2020a) were asked for their views on what client participation entailed, they highlighted the importance of actual decision‐making power, but viewed it as often lacking. This is consistent with Meriluoto's finding (2018, 22) that none of the clients she interviewed, who were participating in development processes and professional driven committees, had ever taken part in actual decision‐making or even been present in environments relevant to decision‐making. Critics of shared decision‐making have also pointed out that the approach mainly works well when decisions concern specific, narrow client's problems, and that no further participation beyond the actual service decision is needed (Treichler & Spaulding, 2017). The SDM model has also been criticized because the professionals do not share the decision‐making power with the client, due to their clinical judgement of the client's ability to make the ‘right’ choice (Treichler & Spaulding, 2017, see Weiste et al., 2020b).

In addition to criticizing the lack of sufficient level of client participation in decision‐making, other researchers have protested the idea of striving towards a symmetric interaction between a professional and a client. Pilnick and Dingwall (2011) noted that professionals’ interactional dominance may be embedded in the medical institution. They argued that this asymmetry serves functional purposes and is necessary for medical enterprise (Pilnick & Dingwall, 2011). Moreover, not only professionals, but also clients constitute and enact asymmetry through their interaction. For the client, following the professionals’ lead, responding to their questions and agreeing with their recommendations might be beneficial to receive the right kind of help for their problem. This means that the professionals usually have the right and duty to take the initiative (e.g. deciding on a diagnosis and recommending an appropriate treatment), whereas the role of the client may be restricted to responding to these initiatives. This type of ‘asymmetry of the initiative’ (Robinson, 2001) has been a topic of a wide range of conversation analysis (CA) studies on decision‐making that have focussed on proposals made by the professionals and the clients’ responses to them during clinical encounters (see Weiste et al., 2020b). The ways in which the professionals respond to initiatives taken by clients have been studied to a much lesser extent, although this would be important for understanding how professionals encourage, or discourage, client participation.

In this article, we consider client initiative in the context of decision‐making in the joint development of social and health‐care services and investigate how professionals respond to clients when they contribute to the discussion by sharing their experiential knowledge. As prior CA research on decision‐making in social and health care has examined clinical encounters in which the decisions have concerned the treatment of an individual client, our study is the first to describe how clients’ contributions are responded to in workshops in which the decisions substantially concern the development of social and health‐care services.

## FROM EXPERIENTIAL KNOWLEDGE TO DECISION‐MAKING POWER

Questions on the relevance of experiential and professional forms of knowledge to the unfolding of interaction may be approached via the CA notion of *epistemics* (Heritage, 2013). In focussing on the ‘knowledge claims that interactants assert, contest and defend in and through turns‐at‐talk and sequences of interaction’ (Heritage, 2013, 370), CA‐based research on epistemics has emphasized the need to consider ‘the in situ interactional characteristics of the exchange of information and the recognition of knowledgeable utterances’ (Housley, 2000, 104). In the field of CA, the entitlements to knowledge that precede and inform the formation and recognition of people's interactional contributions as action are termed their *epistemic status* (Heritage, 2012). Orientations to epistemic status thus constitute a crucial aspect of what is likely to distinguish clients from professionals, but, as with any feature of the social context of interaction, epistemic statuses are verified or challenged in and through the course of interaction (Heritage, 2012).

With regard to epistemic status, clients possess direct, first‐hand knowledge of their own inner experiences, whereas medical professionals enjoy superior general knowledge of medical symptoms and their causes. Clients are thus entitled to their subjective experience as well as to their right to communicate it (Heritage, 2011). As professionals lack direct empirical access to clients’ experiences but are often expected to respond to reports of these experiences, they must demonstrate alternative bases for their claims to understand clients’ experiences (Weiste et al., 2016). The recipients of the accounts of personal experiences (e.g. troubles‐telling and complaints) are expected to show support for the affective stance displayed in the accounts (Stivers, 2008). Such support may be conveyed through, for instance, response cries and parallel assessments, in which the recipient displays access to the teller's experience on the basis of their own earlier experiences (Heritage, 2011). Sometimes, the recipient may seek to affiliate with the teller by generalizing the focus of the account to the extent that they end up questioning the ‘newsworthiness’ of the event or even trivializing the entire experience (Koskinen & Stevanovic, 2022). In contrast, an overly particularized response may sound competitive and even appropriative of the experience (Heritage, 2011). All this suggests that accepting accounts of personal experience is not a straightforward task. Thus, it is important to understand the way in which descriptions of personal experiences—the focal area of client representatives’ expertise—are received by professionals in task‐oriented co‐development workshops.

In co‐development workshops, task orientation has important implications for the interpretation of accounts of personal experiences: these accounts should not only express experiential knowledge but also aid joint decision‐making. In terms of epistemics, joint decision‐making is often motivated by the idea that different participants possess specialist knowledge and expertise in distinct fields. However, such decision‐making requires these pieces of knowledge and expertise to be interactionally transformed into decisions that the participants orient towards binding (Stevanovic, 2012). It is in this respect that participants may be unequally positioned—that is, their capacities to contribute to the emergence of the decision may differ from the outset. Varying degrees of power and authority to establish decisions may be analyzed using the notion of *deontics* (Stevanovic, 2013). The concept of *deontic status* denotes the entitlements that people rely and draw upon when designing their interactional contributions as mutually intelligible actions. However, like its epistemic counterpart, it is reflexively bound to participants’ actions, each contribution in a sequence of interactional events either verifying or challenging the claims of the deontic status implicit in the contribution of the prior participant (Stevanovic, 2013).

Joint decision‐making is a social phenomenon in its own right and is defined as a ‘set of actions, operations and dynamic factors that start with the identification of a stimulus for action and end with a commitment to action’ (Campbell et al., 2019, 296). Joint decision‐making is typically initiated by a participant proposing some future action or event, which implies claiming the deontic right to participate in the ongoing decision‐making activity. However, proposals may also be made more implicitly. Some proposals take the form of evaluations, which, in the activity framework of joint decision‐making, are typically heard as expressions of preferences regarding the content of the decision (Stevanovic, 2012, 790) but may also be received as mere ‘evaluations’ with no deontic relevance. It is thus in and through recipients’ subsequent treatment of proposals that joint decisions emerge—treatment that involves the recipient *acknowledging the deontic relevance of the prior speaker's talk*. This occurs most naturally through accepting responses that pave the way for an agreement and a joint decision, while all other types of response hamper or at least postpone the emergence of a decision. Importantly, in most contexts, no explicit rejection is required to dismiss a decision. Far more frequently, the *de facto* rejection of a proposal is achieved through silence—the proposal is simply ignored or responded with utterances that may be supportive in many ways but fail to acknowledge the deontic relevance of the prior talk (Stevanovic, 2012). Although such deviations from the ‘deontic course of action’ are also possible in response to explicit proposals for future actions and events, they are expected to be even more common in response to implicit proposals, which may take many different forms, such as accounts of personal experiences. This seems to be the case in the co‐development workshops that form the focus of the present study.

This paper pursues two main goals. First, we attempt to reveal the way in which client representatives in workshops on the co‐development of social and health‐care services design their contributions to the ongoing joint decision‐making activity and the responses that their contributions elicit from professionals. Second, we specifically probe the conditions in which professionals treat clients’ accounts of personal experiences as deontically relevant or otherwise laudable.

## DATA AND METHOD

### Research context and materials

Our analysis was based on a dataset of five audio‐/video‐recorded co‐development workshops (15h of interaction). The workshops were held in three large municipal social and health‐care organizations in Finland as a part of the Social and health care professionals as experts on client involvement project. In the first organization, the workshop process took place in a social and health‐care centre and targeted issues concerning the involvement of clients with mental health problems and substance abuse. In the second organization, the process focussed on first‐contact services for elderly and disabled clients. In the third organization, the workshop processes were conducted in two different service units: the rehabilitation ward and the outpatient service unit. The patients in these units were recovering from surgery or undergoing long‐term treatment for a chronic condition (such as diabetes). In all these organizations, client involvement was considered an aspiration for future service delivery, but its realization varied significantly.

The workshops were based on expansive learning theory (Engeström, 1987) and the change‐management workshop method (Virkkunen & Newnham, 2013). In each organization, the co‐development process involved four workshop meetings. Their aim was to (1) create a shared view of client involvement, (2) identify areas requiring improvement, (3) create small developmental experiments to change work practices and (4) evaluate these experiments. In this article, we focus on the third workshops, in which the participants created small developmental experiments to change work practices related to client involvement. These workshops were selected as data as they involved explicit decision‐making on the ideas to be chosen for the actual experiments conducted before the fourth workshop.

In the workshops, the facilitators first outlined the subject and instructed the participants for the group assignment. They divided participants into teams of four, consisting of professionals and at least one client. Their assignment (the same for all the teams) was to generate ideas about the concrete steps required to develop client involvement in the organization. The facilitators asked participants to write down three to five concrete proposals and to discuss the problem that each proposal was to solve. The teams discussed the assignment freely (the facilitators gave only general instructions and watched the time) and made notes. After the separate team discussions, the entire workshop group returned to a facilitator‐guided discussion, in which each team shared their main suggestions. In order to investigate the clients’ initiatives and how they were spontaneously responded to the professionals, the article focusses only on the small group discussions in which the turn‐taking was not allocated by the facilitators.

### Research participants

Each workshop contained approximately 15 participants: eight to 12 professionals (*N* = 38), two to four client representatives (*N* = 9) and two to three facilitators (*N* = 7), totalling 51 participants. As the workshops aimed to develop organizational work practices, the participants were recruited from within the organizations with no research‐based inclusion or exclusion criteria. The professionals represented different occupational groups: nurses (*N* = 17), social workers (*N* = 2), physiotherapists (*N* = 3), development specialists (*N* = 3), service advisors (*N* = 5), department managers (*N* = 7) and one doctor.

The client representatives were recruited from the organization‐based network for clients interested in development activities or by professionals from among their own clients, or from among people known to have been active in prior development activities. Thus, the clients had varying degrees of experience in participating development activities. In the first organization, the two clients had prior experience in using both social and health‐care services for substance abuse types of problems. One of them was trained as an ‘expert‐by‐experience’ (see e.g. Jones & Pietilä, 2020 on different training programmes in Finland) and worked part time as a peer provider. The other was a regular member of a co‐development group in social services. In the second organization, all four client representatives had participated in expert‐by‐experience training and took part in organizational activities, such as giving lectures based on their experiences and guided peer‐support groups. Two of these client representatives were elderly people, one had a chronic physical condition, and the other had a severe physical disability. In the third organization, all three client representatives had a chronic physical condition, and none of them were trained as experts‐by‐experience. They also had less experience in participating in development activities.

The clients received no specific training for the purpose of this research. At the beginning of each workshop process, the role of the clients and professionals were made as equal as possible. For this reason, all the participants introduced themselves by telling the others about their own experiences as clients of the social or health‐care services (thus undermining their roles as professionals and clients). No information on professional titles, training or prior experience in organizational activities was shared. The participants were also instructed to follow the guidelines of dialogical interaction, such as listening to others, expressing opinions and allowing multiple perspectives. However, even though the workshops aimed to promote equal interaction between the clients and professionals, the professionals dominated the interaction: the clients took part in the discussion, but to a lesser extent than the professionals. This was one of the reasons we became interested in what happens during the interaction when client representatives participate and take the initiative to act.

### Ethics

The study was conducted according to the Declaration of Helsinki. Permission to collect data was obtained from the organizations and the Ethics Committee of Finnish Institute of Occupational Health (23 November 2018). Informed, written consent was obtained from all the participants, and they were advised that they could withdraw their consent at any point during the data collection. The anonymity of the participants was ensured by altering names, places and other details that may enable their identification in the text and data excerpts.

### Analysis

The data were analyzed by means of institutional CA, which seeks to explain how social actions contribute to achieving the goals of the institution at hand (e.g. Arminen, 2005). Conversation analysts inductively investigate recordings of naturally occurring interactions to unravel the practices of the interaction through which the meanings of social actions are produced. According to the CA view, social actions are accomplished through adjacent utterances, for instance, sharing an experience elicits affiliation and making a proposal elicits confirmation or rejection (Schegloff, 2007).

In the analytical process, we watched and listened to the recordings several times, and we identified all the sequences of talk in which the client representative took initiating action in the overall context of deciding on future developmental experiments. From 15 h of interaction, we found 47 cases in which the initiating action was either self‐dismissive or self‐promoting. In addition, some more ‘neutral’ actions were taken that did not launch a trajectory towards joint decision‐making. In this article, we focus on only the self‐dismissive and self‐promoting types of instances that we found to be interesting in terms of epistemics and deontics in decision‐making interaction. We analyzed all these instances case by case to specify the nature and variation of the actions, paying attention to their primary interactional function, surrounding context and the ways in which the professionals responded to the clients in their following turns‐at‐talk.

## RESULTS: SELF‐PROMOTION AND SELF‐DISMISSAL AS INTERACTIONAL STRATEGIES

Our qualitative analysis revealed a systematic pattern in our data that linked the clients’ self‐promoting and self‐dismissive turns‐at‐talk to specific types of professionals’ responses. When the clients highlighted the relevance of their experiential knowledge for making decisions, in most cases, their contributions were disregarded by the professionals (*N* = 32). If, however, the clients dismissed their experiential knowledge as irrelevant to the decision‐making activity at hand, this knowledge was often subsequently appreciated by the professionals (*N* = 15).

### Sel‐dismissal as a resource for increasing professional responsiveness

In our data, the clients often referred to their epistemic status as a person with first‐hand experiential knowledge of the organizational practices discussed by the participants. Nevertheless, they dismissed the relevance of this knowledge to the decision‐making activity at hand. In their subsequent turns‐at‐talk, the professionals highlighted the importance of clients’ experiential knowledge to the development of services, even if the client's experiential telling was off the topic (Extract 1). Professionals also highlighted the importance of the clients’ lack of professional knowledge (Extract 2). Lastly, the professionals not only stated that the client's contributions were important, but they also picked up on the clients’ ideas and developed them further, treating them as deontically relevant for the decision‐making activity at hand (Extract 3).

Starting with Extract 1, we show an example of a case in which the client's self‐dismissal invokes appreciation of the client's contribution from the professionals. Prior to Extract 1, one of the professionals had proposed the idea of developing a guide for professionals on the services available in third sector organizations. In the first line, another professional (P1) ponders the benefits of creating an online guide.

Extract 1 (P = professional, C = client representative).


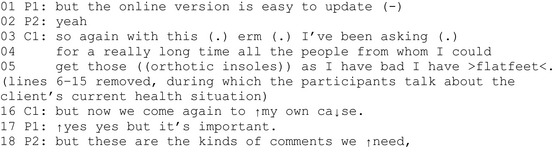


In the middle of planning a development idea, the client representative in line 3 initiates talk about his current health situation. He refers back to the prior conversion making an explicit topic shift. The client's turn is a problem disclosure. He describes his ongoing effort to obtain health‐related services, orthotic insoles, his urgent need for which he explicitly justifies (‘I have flatfeet’, line 5). The client's problem disclosure evokes an affiliation or an attempt to solve the problem as potential next actions (Stivers, 2008), and indeed, the professionals join to discuss the client's personal situation (lines 6–15, not shown in the extract).

Next, in line 16, the client shows an orientation towards his particular experience being irrelevant to the decision‐making activity at hand. He begins with the contrastive conjunction ‘but’, uses the particle ‘again’ and refers to ‘my own case’, which links the client's problem description to his personal experiential domain of knowledge. In line 17, the professional responds by mitigating the client's concern and then validates the client's experience as ‘important’. The other professional adds that the client's experiential account was exactly what was ‘needed’ in service development (lines 18).

In sum, the client dismisses the importance of his personal experience to the decision‐making process. Even though the distance between the client's experiential topic and the topic of the decision‐making activity is quite apparent, the professionals explicitly highlight the importance of the client's contribution. How the client's experiential telling contributes to the decision‐making activity remains, however, undefined.

Extract 2 shows that professionals also highlight the importance of the clients’ *lack of professional knowledge*. Before the extract takes place, the participants have planned a development idea to better inform clients of how their service requests are processed in the system. In the first lines, the facilitator gives general instructions to all small groups and encourages the participants to make as concrete development plans as possible.

Extract 2 (P = professional, C = client representative).


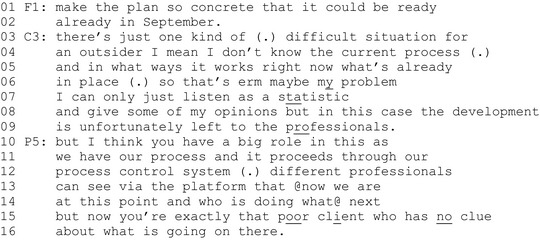


In line 3, the client initiates a turn in which he presents himself as an ‘outsider’ who has no knowledge of the organizational processes. He justifies his argument by claiming to have no knowledge of how the process works (line 5) or what is already in place and functional in the process (line 6). After this, he explicitly states having a problem (line 7). Compared to the previous example, his problem disclosure is not about his personal experience as a patient, but about his role as a client representative in the workshop, based on his lack of access to knowledge on organizational processes, the client can only be a passive assistant who mainly listens (line 7–8) and offers some opinions (line 8). Only the professionals, who have the knowledge of the organizational processes, can participate in the actual development (lines 8–9). To make his point, the client also uses several epistemic disclaimers (‘I don't know’, ‘perhaps’, ‘maybe’ and ‘some opinions’) to dismiss his epistemic status as a co‐developer of the services (Lindström & Karlsson, 2016).

In line 10, one of the professionals responds by highlighting the client's role in the development process. She refers to ‘our’ process and to the way in which each client's process technically proceeds: the professionals have access to the platform and can monitor who does what and how the client's case proceeds in the system (lines 12–14). She then positions the client as a ‘poor client’ in a workshop who has no access to the system and is totally unaware of what is happening there (lines 15–16).

In sum, the client dismisses the importance of his contributions to the service development by referring to his lack of knowledge in the domain of professionals’ expertise. The professionals highlight the importance of the client's lack of knowledge. By virtue of having experience of not knowing the service system, the client's contributions are stated to become relevant for the decision‐making activity at hand.

Lastly, we show an example of a case in which the professionals not only state that the clients’ contributions are important, but also demonstrate it by developing them further, and thus treating them as deontically relevant.

In Extract 3, one of the professionals has suggested the idea of a ‘check‐up call’, which would be made after a client's discharge from the ward. Prior to the extract, the client (C1) has described being discharged and the insecurity she felt at home alone. In the first two lines, the professionals (P1 and P2) affiliate with the client's account, repeating parts of her talk (line 1) and agreeing with her (line 2).

Extract 3 (P = professional, C = client representative).


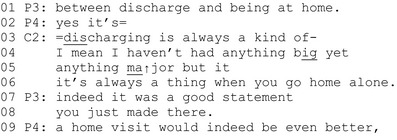


In lines 3–6, the client highlights the importance of discharge from care in the service path of clients, which is exactly the topic targeted by the developmental experiment. She describes being discharged as ‘always a thing’. Such extreme case formulations are an acknowledged means of legitimizing a speaker's claim in interaction (Pomerantz, 1986). In lines 4–5, she refers to her own experience (which she has described earlier in the discussion), and states that she has not suffered ‘anything big yet*’* (line 4), which she then upgrades to ‘anything major’ (line 5), thereby minimizing the severity of her problematic experiences. In this context, this type of minimizing action serves to dismiss the relevance of her account to the decision‐making activity. Here, she seemingly implies that her experiences would not make a ‘good case’ for illustrating the major problems to be focussed on by the developmental experiment. The client takes no direct stance towards the decision on the experiment and leaves the relevance of her interactional contribution open. This is a typical strategy by people in positions of limited authority (e.g. Beach, 2013).

In line 7, the professional responds by affiliating with the client's prior turn (by claiming to recognize the co‐participant's point). She also appreciates the client's contribution by evaluating it as a ‘good statement’. Next, in line 9, another professional (P4) develops the idea further and suggests that they proceed with the idea of a face‐to‐face home visit.

In sum, the client makes an on‐topic contribution and minimizes the relevance of her account to the decision‐making activity. The professionals appreciate the contribution and demonstrate their appreciation by developing the client's idea further, treating it as deontically relevant for the decision‐making activity at hand.

To conclude, when describing their life‐world experiences and participation in the workshops, the client representatives often dismissed their contributions as irrelevant to the decision‐making activity at hand. These self‐dismissive turns invoked appreciation from the professionals. Even if the client's contributions were off the topic, the professionals stated them to be important for the development activity. Sometimes, with more on‐the‐topic contributions, the professionals also demonstrated their importance. They picked up on the clients’ ideas and developed them further. By this way, the professionals increased the deontic relevance of the clients’ experiences, treating them as contributing to the joint decision on the developmental experiment.

### Self‐promotion evoking professional disregard

In our data, the client representatives also sometimes *promoted their experiential knowledge* as relevant to making decisions. In contrast to the self‐dismissive turns‐at‐talk, however, these types of contributions were disregarded by the professionals. Typically, this pattern involved interrupting the client's turn‐at‐talk (Extract 4), or in its most extreme form, ‘sequential deletion’ (Jefferson, 1978)—that is, totally ignoring the client's turn‐at‐talk (Extract 5).

Prior to Extract 4, the professional (P6) had proposed organizing a larger meeting between professionals and clients participating in the organizational development, to share information and ideas on client involvement. In the first lines, the professional suggests that the meeting could involve generating some new ideas on how the collaboration between client representatives and professionals could be further developed.

Extract 4 (P = professional, C = client representative).


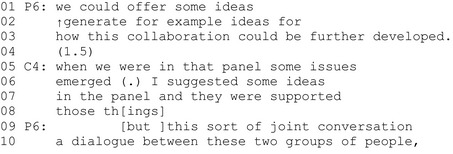


After the professional's turn is completed, there is a short gap in line 4. The client takes a turn and initiates her story about a panel discussion in which she had participated. By referring to a certain moment in time and introducing a new referent (when we were in that panel), the client initiates a new topic and invites the professional to recognize the adequately known referent. The client states that the panel discussion already generated some new ideas, and without directly responding to the professional's proposal, hints that the proposal for organizing a new brainstorming meeting are redundant. She also promotes the importance of her own contribution by stating that she was the one who offered some ideas in the panel (line 6) and these ideas were also supported by the other participants (line 7). By retrospectively describing her own interactional behaviour in the panel in positive terms, the client representative elevates her epistemic and deontic status here and now in the workshop discussion. In line 8, the client is continuing her account when the professional comes in and, overlapping with the client's turn, pursues her own idea about organizing the brainstorming meeting.

In sum, by retrospectively describing her own interactional behaviour in positive terms, the client representative elevates her epistemic and deontic status here and now in the workshop discussion. The professional interrupts the client's self‐promoting turn and continues with her own agenda.

Extract 5 shows another example. In this case, the participants have been planning a developmental experiment for collecting feedback from clients in a small group. Prior to the extract, one of the professionals has proposed that the feedback be collected via email. In the first lines, another professional (P7) opposes this idea and leaves the question open for further discussion (line 4). At this point, the client representative (C5) enters the discussion. This client representative works part time as a peer provider in the given organization, i.e. she leads peer‐support discussions for people with substance abuse‐related problems. The ‘client's mother’ she refers to line 6 is the mother of a client who had participated in a peer discussion with her. The client's mother, who complained about her son being excluded from the services, contacted the client representative to obtain support for her help‐seeking process.

Extract 5 (P = professional, C = client representative).


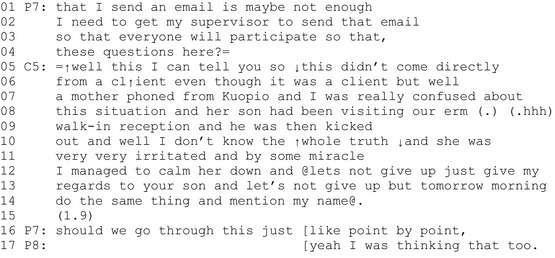


As a response to the decision‐making on the feedback collection method, the client retrospectively describes a particular situation in which she received negative feedback. She provides a trouble description of a situation in which not she (contrary to Extracts 1 and 3) but a client's mother had experienced difficulties. In this case, the complaint about the services is also targeted towards the organization in which the professionals work. By referring to ‘our reception’ (lines 8–9), the client positions herself as one of the professionals. She provides a detailed description of the affectivity of the particular situation (line 7 and 11) and uses reported speech (marked with@) to dramatize the situation (lines 12–14). By describing emotions and using reported speech, the client brings ‘the voice of the experience’ to the discussion. In a similar way to that in Extract 4, she also elevates her epistemic and deontic status here and now by retrospectively describing her own interactional behaviour in the feedback situation in positive terms.

The client's account is ignored by the professionals. During her description, the other participants produce no vocal feedback, and upon completion of her account, all the recipients remain silent (line 15). Next, the professional (P6) returns to the workshop agenda and, by referring to the assignment sheet, proposes that the participants begin working through it. The other professional agrees, stating that this was something that she was also considering (line 17).

In sum, Extract 5 demonstrates how the client brings her experiential knowledge into the discussion and elevates her epistemic and deontic status by retrospectively describing her own interactional behaviour in the feedback situation in positive terms. The client's turn is ‘sequentially deleted’—that is, totally ignored by the professionals.

To conclude, the clients elevated their epistemic and deontic status by describing their prior interactional experiences in positive terms. By this way, they positioned themselves as knowledgeable and capable workshop participants. Their contributions were, however, systematically disregarded by the professionals. Thus, paradoxically, in order to gain epistemic and deontic relevance for their views, the client representatives withdrew from their position as experts of experience.

## DISCUSSION

This paper examined the ways in which client representatives in workshops for the co‐development of social and health‐care services designed their contributions to an ongoing joint decision‐making activity and investigated the types of response their contributions elicited from the professionals present. We found two recurring interactional patterns. The first demonstrated that when the client representatives dismissed their experiential knowledge as irrelevant to the decision‐making activity at hand, their accounts invoked appreciation from the professionals. This pattern is in line with prior research, which has shown that epistemic tensions among participants, especially in client–professional interaction in health care, are typically handled by clients using epistemic disclaimers (Lindström & Karlsson, 2016). By using such disclaimers, clients can mark their awareness of the tensions caused by taking initiatives typically reserved for professionals (Lindström & Karlsson, 2016). In our data, easing these epistemic tensions seemed to increase the professionals’ responsiveness to the clients’ actions. In cases in which the client's contribution could otherwise be treated as ill‐timed or off topic during the interaction (see Extract 1), this type of professional response was also sensitive to the need to avoid the client losing ‘face’ (Goffman, 1961). Sometimes, the professionals also made public their reasoning for *why* a client's contribution could be considered commendable (Extract 3). In this way, the professionals increased the deontic relevance of the clients’ experiential accounts, treating them as a contribution to the decision‐making on the developmental experiment.

The second interactional pattern showed that when the client representatives promoted their experiential knowledge as relevant to making decisions, their accounts were ignored by the professionals. By remaining silent (Extract 5) or interrupting the client and continuing their own agenda as if the client's turn had never occurred (Extract 4), the professionals were able to reject the client's idea without explicitly disagreeing with it (Stevanovic, 2012). Although ignoring the turn in this way may protect the client's face compared with overtly conflictual actions, such as rejections, it nevertheless disaffiliates with the client's description of their personal experiences (Stivers, 2008) and undermines the social solidarity between the participants (Lerner, 1996). Indeed, people are generally quite sensitive to a lack of recipient affiliation in response to descriptions of personal experiences, and the absence of this affiliation has been associated with increases in physiological arousal and indicators of stress (Peräkylä et al., 2015).

One possible explanation for the professionals’ disregard of the client representatives’ contributions could be their unwillingness to share power. Studies on participatory governance in which citizens participate in the development of communal services together with public servants have shown that their presence is often considered a token gesture to fulfil the obligation of involving citizens (Arnstein, 1969). Thus, it seems likely that public servants have no real desire or intention to change power relations to enhance the say of citizens in the co‐development of services (e.g. Leino, 2006). Rather, civil servants simply wish citizens to agree with them and align with the decisions taken prior to the joint development situations (see also Lewis, 2014). Our workshop data showed no such pre‐made decisions concerning the developmental experiments, and thus, it was possible for the professionals to take the clients’ contributions into consideration in a meaningful way. However, the fact that the professionals still often ignored the clients’ contributions points towards considerable challenges in their power relations: greater client involvement may be experienced as a threat to professional boundaries and competencies (e.g. Higgins, 1994).

Although the strength of this study is its detailed analysis of interactional patterns in naturally occurring workshop discussions, it also has certain limitations. We have described reoccurring interaction patterns in our data, which point to systematically different ways in the participants’ responses to the initiating turns by the professionals, on the one hand, and to those by the clients, on the other hand. But of course, all claims about causality are only interpretations of the patterns. As for single data examples, this is even more the case—and an interactional outcome may, of course, be in principle—attributed to any feature of the case, which deviates from another case. It is throughout possible that clients’ initiating turns exhibit systematically a somewhat worse fit with the ongoing interaction than the otherwise similar initiating turns by the professionals. However, after going through our entire data specifically from this viewpoint, we could not make this type of a conclusion. Another obvious limitation is the relatively small number of participants taken from a very specific interactional context, which naturally limits the generalisability of our results. For instance, a range of practitioners were involved in the interactions, but we could not make a quantitative point on the possible differences in their receptiveness to the clients. Possible differences between professional groups, for instance, remain topics for future research. Furthermore, the client representatives who participated in the workshops were recruited by the professionals from among their own current clients and clients who had already participated in different types of development interventions. Thus, there were considerable differences in their skills and competences (see Aronson, 1993). This offers intriguing opportunities for future research. Some prior research has suggested that client participation in different types of development workshops requires a degree of ‘proto‐professionalism’, i.e. formal training that helps the clients act as ‘experts’, in order to be taken seriously by the professionals present (e.g. Meriluoto, 2018). Our findings, however, raise the question of whether it is exactly these types of expressions of expertise that the professionals treat as problematic, hindering the possibilities for client experiences to be taken into consideration in decisions on the development of services. Thus, more comparative research of trained and ‘ordinary’ clients is needed to determine the role of training the ‘experts‐by‐experience’ for actual participation in the development workshops.

While equality, participation and social inclusion are buzzwords in today's political discourse, joint decision‐making may be regarded as the basic locus of participation in society. This cultural ideal is also reflected in the development of social and health‐care services—most prominently, in the mere fact that the types of co‐development workshops analyzed in this study were organized in the first place. However, as our study has shown, it is one thing to organize situations in which joint decision‐making can occur and another to achieve equal levels of participation and establish genuine joint decisions in practice. Indeed, as has been demonstrated in micro‐level analyses of joint decision‐making interactions in various contexts (Asmuss & Oshima, 2012; Stevanovic, 2013; Weiste et al., 2020b), establishing joint decisions is a truly complex endeavour in which the social world, with its entire web of social relations of status and rank, is constructed, negotiated and manifested in the concrete ways in which proposals are formulated and responded to others. Arguably, it is a person's understanding of self that is at stake every time they make a proposal—particularly proposals based on personal experience.

## AUTHOR CONTRIBUTIONS

**Elina Weiste:** Conceptualization (equal); Data curation (lead); Formal analysis (equal); Funding acquisition (lead); Investigation (equal); Methodology (lead); Project administration (lead); Resources (lead); Validation (lead); Writing – original draft (equal); Writing – review & editing (equal). **Melisa Stevanovic:** Conceptualization (equal); Data curation (equal); Formal analysis (equal); Investigation (equal); Methodology (equal); Validation (equal); Writing – original draft (equal); Writing – review & editing (equal). **Lise‐Lotte Uusitalo:** Conceptualization (equal); Data curation (supporting); Formal analysis (equal); Investigation (supporting); Writing – original draft (supporting); Writing – review & editing (supporting).

## APPENDIX 1 Transcription symbols

[] Overlapping talk.

(.) Silence measured in seconds and tenths of a second.

word accented sound.

‐ Cut‐off of preceding sound.

? Rising intonation.

, Level intonation.

. Falling intonation.

>text<Speech delivered more rapidly than usual.

↑↓ Rising/falling pitch.

(.hhh) Audible inhalation.

@text@ Animated voice.
